# Inhibitory Effect of Ionizing Radiation on *Echinococcus granulosus* Hydatid Cyst

**DOI:** 10.3390/diseases7010023

**Published:** 2019-02-18

**Authors:** Yumin Zhao, Weifeng Gui, Yishu Zhang, Gang Mo, Dayu Li, Shigui Chong

**Affiliations:** Department of Nursing teaching and Research, School of Basic Medicine, Guilin Medical University, Guilin 541004, Guangxi, China; zymcsg@sina.com (Y.Z.); 17779897914@163.com (W.G.); xianbaodandan@163.com (Y.Z.); mogang@glmc.edu.cn (G.M.); happiness1993@foxmail.com (D.L.)

**Keywords:** inhibitory effect, ionizing radiation, *E. granulosus*, hydatid cyst, zoonosis

## Abstract

**Background:** Heavy ion radiation has more advantages than traditional radiation therapy in the treatment of cancer, mainly because of its superior biological effects. However, there is currently no reliable evidence that heavy ion radiation can induce cell death in hydatid cysts at the cellular and molecular level. In addition, we believe heavy ion therapy could be a potential alternative approach for the treatment of hydatid cysts. **Methodology/Principal Finding:** The hydatid cysts and protoscolices were obtained from an experimentally infected KunMing mice. LD50 was used to evaluate the death of the protoscolex. The cellular and ultrastructure of the parasites were observed under light and electron microscopes, the damage and copy numbers of mitochondrial DNA (mtDNA) were decided by QPCR. The apoptosis was evaluated by the expression and activity of caspase3. Dose-dependent ionizing radiation induced damage to the initial mtDNA. Echinococcosis cyst after ionizing radiation showed sparse cytoplasm, disorganized and clumped organelles, huge vacuoles, and villus deletions. The kinetic of DNA repair activity after X-ray irradiation was faster than those after carbon-ion irradiation. High doses of carbon ion radiation caused irreversible attenuation of mitochondrial DNA. Cysts showed obvious reduction in size after radiation. Carbon ion radiation was more effective than X-ray radiation in inhibiting hydatid cysts. **Conclusions**: These studies provide evidence that heavy-ion radiation can cause the extinction of hydatid cysts in vitro. The carbon-ion radiation is more advantageous than X-ray radiation in suppress hydatid cyst.

## 1. Introduction

Echinococcosis/hydatidosis is caused by larvae of *Echinococcus granulosus (E. granulosus)*, commonly found in Europe, China and Siberia. This is a zoonosis with a high lethality. The most common intermediate hosts of *E. granulosus* are dogs. However, sheep and human become infected mainly by oral intake of *E. granulosus* eggs released by dogs. The current treatment used for hydatid cysts is surgery combined with chemotherapy using albendazole and/or mebendazole. However, surgical treatment does not work when the cysts are in a critical position or in multiple tissues and organs. In this case, PAIR (puncture-aspiration-injection-reaspiration), combined with chemotherapy, becomes an alternative option of treatment [1]. High-energy electromagnetic waves (such as X-rays) are a new treatment found in current new researches. Hydatid cyst consists of germinal cells and stratum corneum cells and has millions of protoscolices within it. In vitro and in vivo studies have found that X-ray irradiation can significantly induce the death of protoscolex and inhibit the growth of cysts [2,3]. A recent case study presented radiation therapy as an alternative treatment modality for hydatid disease of the chest wall after medical and surgical therapy failure [4]. However, X-rays are not a good choice for treating Echinococcosis cysts, because most of the *E. granulosus*, localized in the liver and lungs, and the liver and lung tissue are not tolerant of X-ray radiation. Therefore, treatment does not work well, and neither is safe [5,6,7]. In the treatment of small cell lung cancer and hepatocellular carcinoma, heavy-ion radiotherapy in the local control and survival have significant advantage, and has proven to be an ideal method of treatment [8,9]. High-energy beams of charged nuclear particles have significant advantages in the treatment of the deep-seated hydatid cysts compared to the conventional megavolt photon therapy. Heavy-ion radiation has a well-defined range, smaller lateral beam diffusion and enhanced biological effects, so heavy-ion radiation can be an effective way to treat hydatid cyst.

There are currently no in vitro reports of cellular and molecular effects on hydatid cysts with heavy-ion radiation. Here, we report for the first time the carbon-ion irradiation induced hydatid cysts cell death.

## 2. Materials and Methods

### 2.1. Ethics Statement

All animal experiments described have been conducted according to the Guideline on the Humane Treatment of Laboratory Animals stipulated by the Ministry of Science and Technology of the People’s Republic of China (MOST) and were approved by the Institutional Animal Care and Use Committee (IACUC) of Institute of Modern Physics, CAS for the use of laboratory animals. The Regulations for the Administration of Affairs Concerning Experimental Animals Committee (1988.11.1) are affiliated with Institute of Modern Physics, CAS.

### 2.2. Sample Collection

Hydatid cysts of *E. granulosus* samples were freshly isolated from the liver of secondary infected female mice. The first infection was a sheep naturally infected with *E. granulosus* in Qinghai, China. The cysts were rinsed repeatedly with sterile saline several times, then cultivated with RMPI 1640 medium at 37 °C in a 5% CO_2_ incubator.

### 2.3. Radiation Procedure

X-ray was generated with an X-ray’s machine (FAXITRON RX650, Faxitron, Washington, DC, USA) operated at 130 keV. An exposure-rate meter (AE-1321 M, Applied Engineering Inc, Tokyo, Japan) was used for the dosimetry. The dosage rates were 1.3 Gy/min. Carbon-ion irradiations were performed at room temperature at the Heavy Ion Accelerator Center (HIRFL) at the Institute of Modern Physics (Lanzhou, China) with 300 MeV/n carbon ions; the LET value for carbon ions was 40 KeV/um. The dose rates were 1 Gy/min. Compared with X-ray, Carbon-ion irradiations have higher energy levels, stronger penetrability and no damage to normal cells.

### 2.4. LD50 Assay

The hydatid cysts LD50 were determined according to the death of protoscolex after radiation administration. After 24 h of irradiation, the viability was observed for of protoscolecess with a microscope. Randomly selected ten areas were chosen to determine the corresponding numbers of viable/non-viable under 10× magnification microscopy (Nikon Corporation, Tokyo, Japan).

### 2.5. Light and Electron Microscopy

After they had been irradiated with the two different radiation types, cysts were fixed in 4% glutaraldehyde (24 h) and 1% osmium tetroxide sequentially dehydrated with acetone gradient, and embedded in Epon 812 epoxy resin (Hexion Specialty Chemicals, Inc, Shanghai, China). The 60-nm sections were cut with an ultrathin microtome, stained with uranium and lead electron stains, and observed under the transmission electron microscopy (TEM) (ZCJCE, Guangzhou, Guangdong, China). Cysts were also observed under light microscopy according to standard protocol.

### 2.6. Immunoblotting

Western blotting protocol was used according to standard protocol. In short, the protoscoleces were dissolved in the buffer containing 50 mmol/L Tris at pH 7.4, 50 mmol/L NaCl, 0.1% Triton X-100, 0.1% SDS, 0.3 mmol/L sodium orthovanadate, 1 mmol/L dithiothreitol, 10 mg/L leupeptin, and 5 mg/L aprotinin. Protein concentrations of lysates were determined using a BCA protein assay kit (Pierce, Rockford, IL, USA). An aliquot of each extract (40 µg protein) was transferred to a PVDF membrane by an SDS-polyacrylamide gel. Membranes were blocked with 10 ml TBST containing 0.5 g FBS at room temperature for 2 h, followed by incubation with antibodies against Caspase-3 (Biosynthesis Biotechnology, Beijing, China) at 4 °C overnight. First, the membranes were rinsed with TBST for 30 min, and, after an hour of incubation at room temperature, an appropriate amount of HRP-conjugated secondary antibody was added to the membranes. Then the membranes were rinsed three times with TBST for 15 min each time. Reactive proteins were visualized using a chemiluminescence kit (Santa Cruz Biotechnology, Santa Cruz, CA, USA) according to the manufacturer’s instructions.

### 2.7. Caspase-3 Activity Assay

Detections were performed according to a previous description using the caspase-3 activity colorimetric assay kit instruction (Beyotime Institute of Biotechnology, Nantong, Jiangsu, China) [10]. Briefly, 3 mg samples were first added to 100 μL lysis buffer, kept on ice for 15–20 min, then centrifuged at 4 °C, 17,000× *g* for 15 min. According to the kit’s instruction, the supernatants were harvested and added into the reaction system on an assay plate with the control group; incubated the plates at 37 °C for 15 h and detected with a microplate reader for the absorbance at 405 nm (A405). First, the concentration of PNA was calculated from the pNA standard curve and template A405. Second, the activated caspase-3 in samples was catalyzed when colorless substrate Ac-DEVD-pNA turned into yellow pNA; finally, the activity of caspase-3 in samples was deduced based on the pNA concentration.

### 2.8. MtDNA Damage Assay

mtDNA damage of long PCR sequences was estimated by the GeneAmp XL PCR kit (PerKin-Elmer, Boston, MA, USA). Quantitative long PCR was performed in an Eppendorf Master cycler PCR system (Eppendorf, Hamburg, Germany). The PCR in the exponential phase was ensured and the PCR cycle test was performed. In short, DNA-directed RNA polymerase II (rpb2) as a reference for nuclear DNA copy number, which is a highly conserved single copy nuclear gene. Cytochrome c oxidase subunit II (COX2) is used as a mtDNA copy number reference, which is an essential part of encoding mitochondrial Electron Transport Chain (ETC). PCR was initiated with a 75 °C hot-start addition of the polymerase then the steps below were followed to continue the experiment: the initial denaturation was performed at 94 °C for 1 min followed by 25 cycles of large fragments or 20 cycles for small fragments, then 15 min extension at 68 °C. A final extension at 68 °C was performed for 10 min at the completion of the profile. Aliquots of each PCR product were separated on a 1% vertical agarose gel and electrophoresed in TBE for 4 h. Quantitative analysis of the gel with was performed with FluorChem FC2 (Alpha Innotech corporation, Santa Clara, CA, USA) and digital photographs of the gel were taken. The relative level of amplification of the large fragments of mtDNA (8761 bp) were compared to quantify the DNA damage, normalizing this to the amplification of smaller (126 bp) fragments.

### 2.9. Statistical Analysis

Data were obtained from at least three independent experiments and statistically analyzed. Data are expressed as means ± SD. Student’s *t*-test program in Microsoft Excel was used to detect statistical significance. *p* < 0.01 means that there is statistical significance.

## 3. Results

### 3.1. Effect of Ionizing Radiation on Hydatic Cysts Survival

**Figure 1:**
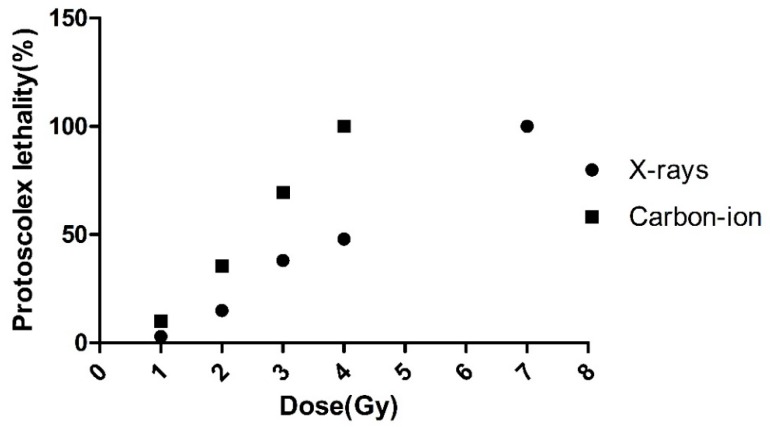
Percent lethality versus radiation dose. The data are fitted to a sigmoidal dose response function: y = a + b/(1 + exp(−(x(−c)/d)), where y is the percent lethality, x is the dose in Gy, respectively.

The reduction of protoscoleces activity (data not shown), and the decrease in vitality became visible as early as 3 h after of ionizing radiation. In the IR-treated cultures, the viability of the original protoscolex was significantly lost after 24 h (*p* < 0.05). At 60 Gy X-ray or 30 Gy carbon after the ionizing radiation, the protoscolex mortality was 100%. There was an increased pronounced dose dependent lethality after ionizing radiation compared with X-rays, as presented in Figure 1.

The LD50 was 15.5 Gy for carbon-ion and 28.5 Gy for X-rays, respectively. The LD50 of carbon-ion irradiated cysts was significantly less than that for X-ray radiation.

### 3.2. MtDNA Damage Repair and mtDNA Copy Number in Irradiated Hydatid Cysts

**Figure 2:**
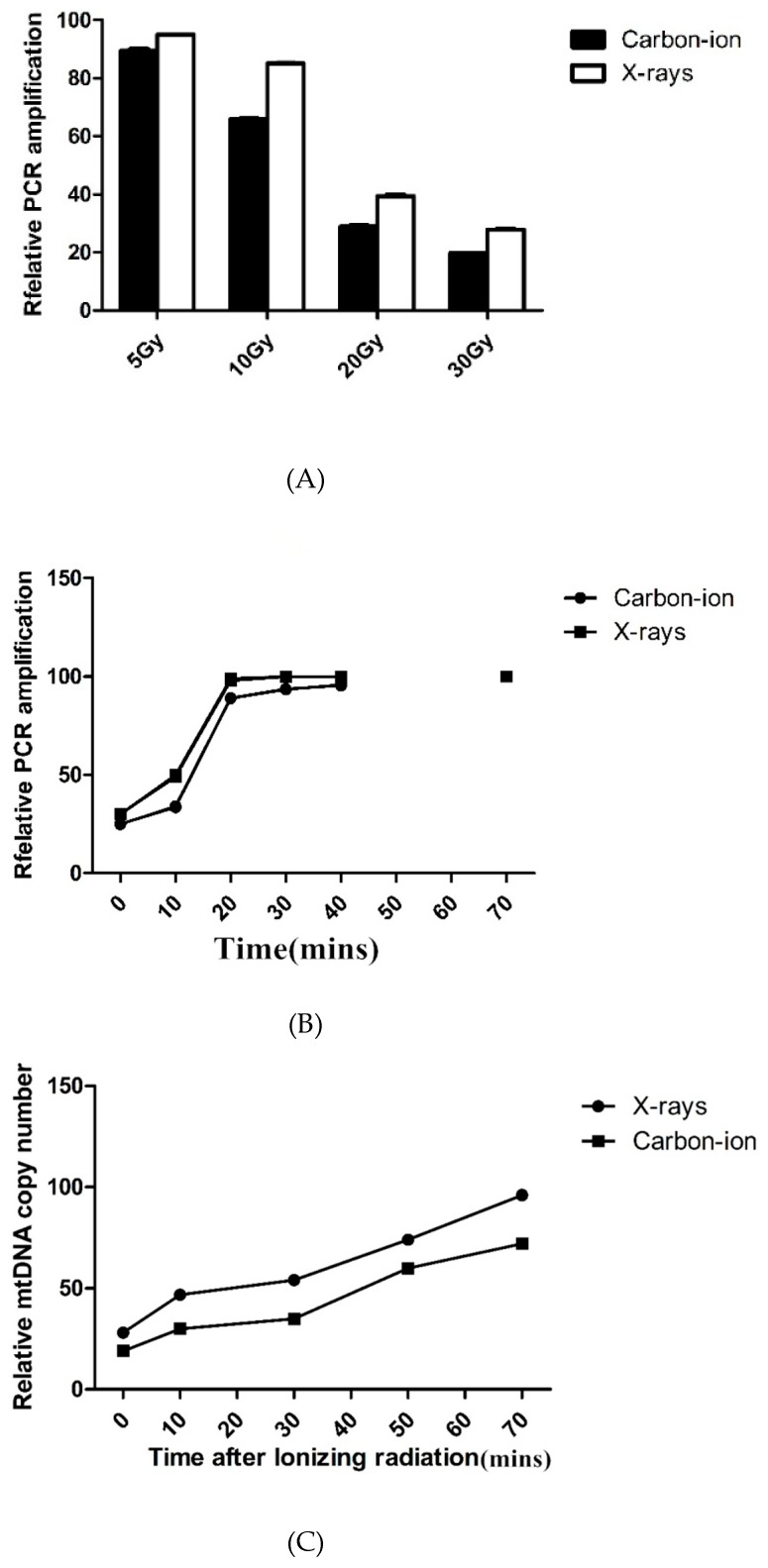
(**A**) DNA damage repair and mtDNA copy number in irradiated hydatid cysts. Quantification of mtDNA (mitochondrial DNA) damage by long-PCRamplification of total DNA isolated from *E. granulosus* hydatid cysts after 5–30 Gy X-rays or carbon-ion radiation. MtDNA damage was indicated by reduced PCR amplification. (**B**) DNA damage repair and mtDNA copy number in irradiated hydatid cysts. Repair kinetics of mtDNA in *E. granulosus* hydatid cysts within 72 h after 30 Gy X-rays or carbon-ion radiation. (**C**) DNA damage repair and mtDNA copy number in irradiated hydatid cysts. Quantification of mtDNA copy number by real-time PCR.

There was a dose-dependent correlation between carbon-ion and X-ray radiation on mtDNA damage. Compared with X-rays, mtDNA damage caused by carbon-ion irradiations at the same radiation dose was significantly higher (Figure 2A); yet both groups of result demonstrated mtDNA have the same effective repair (Figure 2B).

Even though mtDNA damage was completely restored within 8 h of irradiation, we found that the COX2 signal was significantly reduced 4 h after irradiation, indicating a significant decrease in mtDNA copy number. The mtDNA copy number was quantified by real-time PCR, and the results showed that the mtDNA copy number decreased by up to 30% after radiation and gradually recovered after 24–48 h (Figure 2C).

### 3.3. Morphological Alterations of Irradiated Parasites

**Figure 3:**
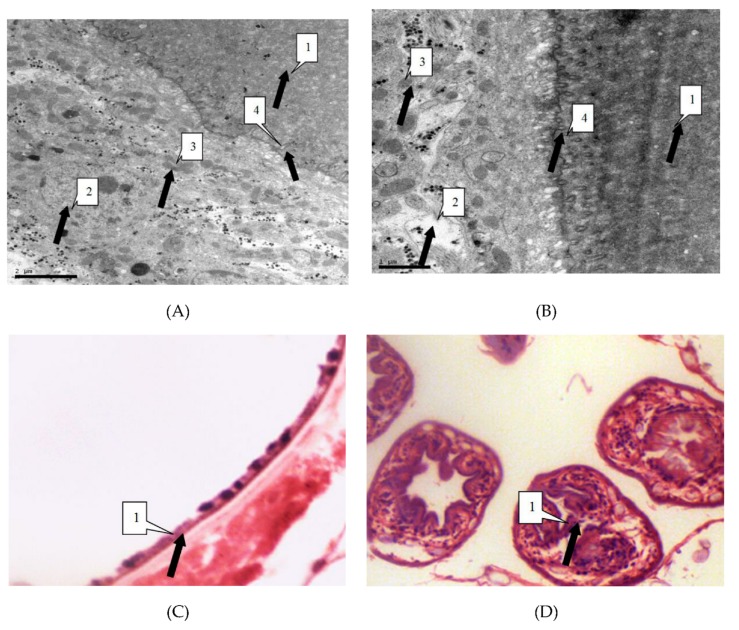
(**A**) Electron microscope magnified 10,000 times. As shown by the arrow, cells of the germinal layer (2) down (3) into the laminated layer (1). Organelles such as cells of the germinal layer (4) mitochondria and Golgi complex show basic integrity. No devoid of villi. (**B**) Electron microscope magnified 20,000 times. The Figure B describes similar changes to the Figure A. (**C**) The light microscope magnified 400 times. As shown by the arrow, the germinal layer (1) and laminated layer (1) remained intact. (**D**) The light microscope magnified 1,000 times. As shown by the arrow, the form of protoscoleces remained intact.

We observed the parasites of the irradiated group and the control group by thin section electron microscopy 24 h after irradiation, in order to study the influence of radiation on hydatid cyst cells. Observation showed that in the control group cysts, the germinal cells are intact, under the villi beneath the cuticle. In addition, organelles such as mitochondria and Golgi apparatus are visibly arranged around the cells. Cells in the irradiated group showed obvious abnormalities, including sparse cytoplasms, absence of organelles such as mitochondria and Golgi, and lack of villi. These were the manifestations of injured or dead cells (Figure 3).

**Figure 4:**
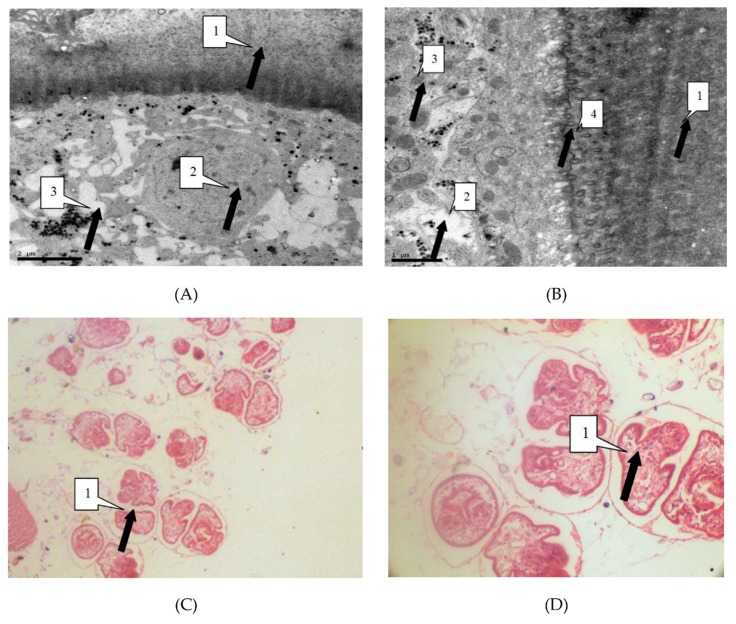
(**A**) Electron microscope magnified 12,000 times. As shown by the arrow, the villi that the germinal layer (1) extended to the laminated layer (2) has disappeared (100/100). Organelle such as cells of the germinal layer (1) mitochondria and Golgi complex lost basic integrity. There was a large number of vacuoles (3) in the cells of the germinal layer (100/100). (**B**) described equally to the Figure A. Electron microscope magnified 25,000 times. (**C**) The light microscope magnified 400 times. As shown by the arrow, the protoscolexs were valgus (1). (**D**) The light microscope magnified 1000 times. The Figure D described equally to the Figure C.

Light microscope showed that the control group of cell morphology was complete and clear. The protoscolex, germinal layer, and cuticles were intact and visible. In the X-ray irradiated group, abnormal protoscolex and detachment of germinal were detected in the layers from cuticles. In the carbon-ion irradiated group, protoscolex contraction, loss of suction cups, and scolex hooks were extensive (Figure 4).

### 3.4. Radiation Induced Hydatid Cysts Apoptosis

**Figure 5:**
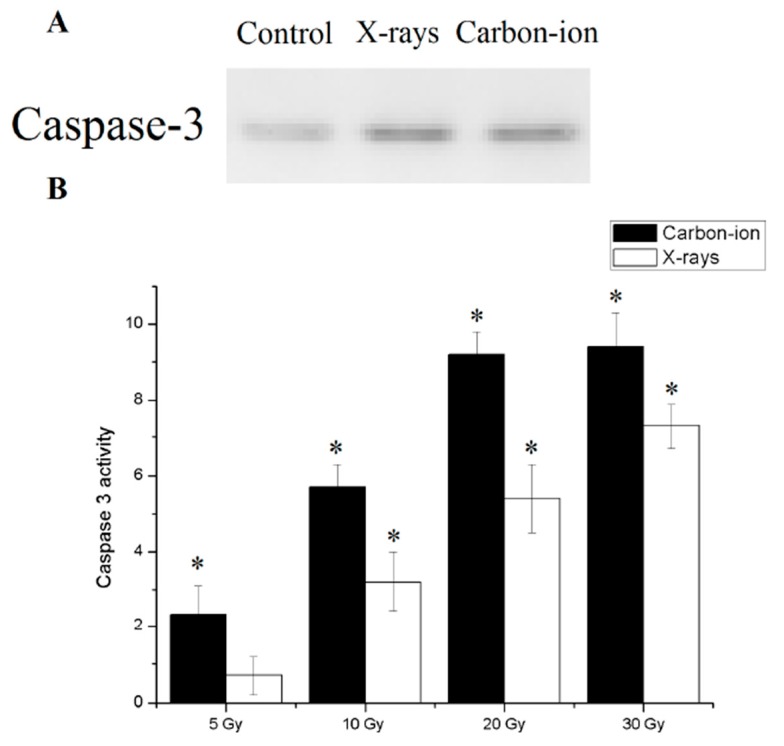
Apoptosis in 30 Gy X-rays and carbon-ion irradiated *E. granulosus* hydatid cysts. (**A**) Caspase 3 expression in hydatid cysts with and without 30 Gy ionizing radiation. (**B**) Caspase 3 like activity was measured as the difference in pNA production between the samples with and without ionizing radiation. Error bars represent the SD, each done in at least triplicate. * Statistical significant at *p* < 0.01.

Since the metabolic pathway of programmed cell is currently unknown in *E. granulosus*, caspase3, an effector molecule common to all known metabolic routes of apoptosis procedure was used as indicator of apoptosis. After 30 Gy of X-rays or carbon-ion radiation, the detected caspase 3 in the hydatid cyst was used to indicate the apoptotic index (Figure 5A).

Finally, Caspase 3 activity was significantly higher in the irradiated cysts than in the control group. At the same dose, the activity of caspase 3 after carbon ion irradiation was higher than that of X-ray radiation. Caspase 3 activity reached a plateau after 30 Gy radiation exposure (Figure 5B).

## 4. Discussion

In this research, we first demonstrate at the cellular and molecular level the effects of high-dose X-rays or carbon-ion radiation on *E. granulosus* hydatid cysts. Exposure to high doses radiation causes the attenuation of hydatid cysts. The morphological changes of hydatid cysts after radiation were similar to other reports using drugs [10,11], confirming the radiation on the hydatid cyst inhibition.

It is possible that the mitochondrial repair capacity of the hydatid cysts exceeded the upper limit, so that a significant decrease in mtDNA copy number indicated that the degradation process of mtDNA became profound with increase oxidative damage. The persistent depletion in mtDNA content appears to be a direct consequence of active mtDNA degradation and may be the basis to the “persistent mtDNA damage” reported in several studies [12,13]. The profound mtDNA damage and degradation then could lead to mitochondrial dysfunction and persistent oxidative stress [14]. *E. granulosus* major source of energy is carbohydrates which can be catabolized by anaerobic respiration or two complementary anaerobic pathways. Radiation can induce mitochondrial dysfunction, and, due to its complex parasitic lifestyle, can lead to the death of hydatid cyst cells and inhibit cyst growth.

It has been reported that high levels of apoptosis are involved in hydatid cyst infertility in *E. granulosus* hydatid cysts [15,16]. Therefore, the modulator of hydatid fluid, which leads to lymphocytes apoptosis, is one of the mechanisms of hydatid cysts survival [17]. Hanhua, et al. reported that H_2_O_2_ and dexamethasone could induce the cellular apoptosis of protoscolices [18]. However, there is no report on the oxidative stress induced apoptosis in *E. granulosus*. We found that ionizing radiation, such as X-rays and carbon-ion radiation, could effectively induce apoptosis in *E. granulosus* hydatid cysts, which may be caused by exacerbated oxidative stress [10].

Compared with the traditional physical and biological therapy, heavy-ion radiation therapy has high local control rates, while compared with photo and proton radiation therapy, heavy-ion radiation therapy is relatively low toxicity [19,20]. Our results also show that carbon-ion radiation causes more damage to hydatid cysts than X-rays. However, the side effects of heavy-ion radiation therapy should also receive the same attention. Severe chronic complications have been reported in patients who received high dose heavy-ion radiation for esophageal cancer [20]. Further studies should provide further *in vivo* experimental data on the treatment of hydatid cysts with heavy-ion radiation.

At present, our research results provide a theoretical basis for exploring the application of radiotherapy as a non-surgical treatment in the treatment of parasitic diseases. In addition, we report that carbon-ion radiation is more effective than X-rays in the treatment of hydatid cysts, as this treatment may be more suitable candidate for hydatid disease.
